# Visible Epiglottis in an Older Adult: A Potential Indicator of Presbyphagia

**DOI:** 10.1002/ccr3.73323

**Published:** 2026-08-09

**Authors:** Miki Yamada, Yosuke Iijima, Kenta Ushikubo, Shuto Mochizuki, Shunsuke Hino, Norio Horie, Takahiro Kaneko

**Affiliations:** ^1^ Department of Oral and Maxillofacial Surgery, Saitama Medical Center Saitama Medical University Saitama Japan; ^2^ Department of Oral and Maxillofacial Surgery II Nihon University School of Dentistry Tokyo Japan

**Keywords:** aspiration pneumonia, epiglottis, older adult, presbyphagia, swallowing dysfunction

## Abstract

Macroscopic visualization of the epiglottis during routine oral examination is uncommon but may suggest swallowing dysfunction in older adults. This finding may be associated with age‐related changes in tongue‐base support and may warrant further assessment of swallowing function.

## 1 Introduction

Aspiration pneumonia remains a leading cause of mortality and morbidity in older adults, and its prevention is closely associated with the maintenance of adequate oral health and function. Accordingly, the importance of oral health is well‐recognized in geriatric facilities, leading to the active implementation of oral health care for residents [1, 2]. Dental hygienists and dentists who provide continuous care are uniquely positioned to observe subtle but critical changes in the oral and pharyngeal environments.

The epiglottis is a leaf‐shaped elastic cartilage at the entrance to the larynx. Its primary function is airway protection; during the pharyngeal phase of swallowing, the hyoid bone and larynx rise while the epiglottis reflexively folds backward to seal the glottis [3]. Under normal conditions, the epiglottis is located posterior to the base of the tongue, making it nearly impossible to visualize during a routine intraoral examination.

In this report, we present an uncommon case in which the upper epiglottis was visible to the naked eye during maximum tongue protrusion in a 94‐year‐old patient. We discuss the possible clinical significance of this finding and its potential association with presbyphagia and aspiration pneumonia.

## 2 Case Report

The patient was a 94‐year‐old woman residing in a nursing home with a medical history of hypertension, cerebral infarction, osteoporosis, colon cancer, glaucoma, and mild cognitive decline. Her medication included amlodipine besilate, carvedilol, alfacalcidol, aspirin, brexpiprazole, tiapride hydrochloride, and magnesium oxide. She was edentulous and wore complete dentures. She was 145 cm tall and weighed 37.9 kg.

**FIGURE 1:**
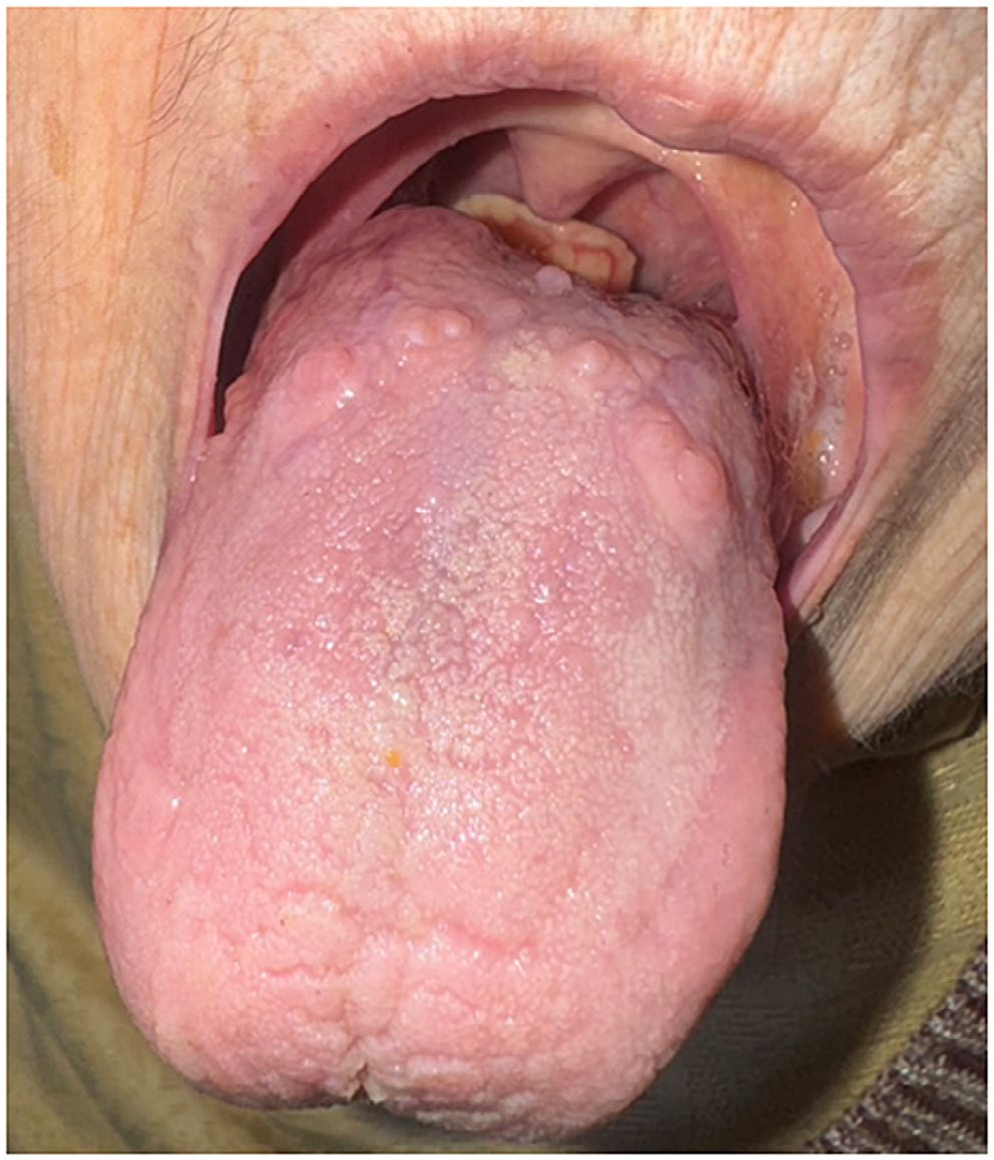
The upper part of the epiglottis is seen at the base of the tongue during maximum tongue protrusion. The observed structure shows a yellowish‐white border, a reddish interior, and a more yellowish area on the left side.

During a routine oral hygiene care session, a dental hygienist noticed a protrusion at the base of the tongue that had not been seen during the previous care visits. No foreign body sensation or pain was noted (Figure 1). The hygienist photographed the finding and reported it to the attending dentist. Although the structure was not immediately visible upon the dentist's re‐examination, the photograph prompted a consultation with our oral surgery department.

The photograph showed a transverse structure approximately 30 mm in length located posterior to the tongue base and below the uvula. It exhibited a yellowish‐white border and a reddish interior with a yellowish area on the left side, characteristic of the upper margin of the epiglottis.

A targeted re‐examination revealed a low‐positioned tongue. While tongue tip movement and protrusion were preserved, the tongue base appeared significantly depressed.

### 2.1 Differential Diagnosis

The patient consumed a soft chopped diet and had no history of coughing or choking during meals. There was no reported recent weight loss. Although she maintained oral intake without overt swallowing difficulty in daily life, the Repetitive Saliva Swallowing Test (RSST) yielded 3 swallows in 30 s [4]. While this met the conventional cutoff value (abnormal ≤ 2 swallows), it represented a borderline result that raised concern for possible early swallowing dysfunction.

### 2.2 Outcome and Follow‐Up

Three months after the documentation of the visible epiglottis, the patient was hospitalized due to acute fever and diagnosed with aspiration pneumonia. Although the pneumonia was successfully treated with antibiotics, the three‐month hospitalization resulted in a marked decline in her physical and cognitive status. Due to the significant reduction in her activities of daily living (ADL), returning to her previous nursing home became unfeasible, and she is currently being transferred to a facility requiring more intensive nursing care.

## 3 Discussion

This case report describes an uncommon clinical observation in dental practice: the macroscopic visualization of the upper epiglottis during maximum tongue protrusion in an older adult.

Among dental professionals, direct visualization of the epiglottis during routine oral examination is rarely encountered. However, in anesthesiology, the Mallampati classification (modified by Ezri et al.) for assessing the difficulty of endotracheal intubation includes Class 0, defined as visualization of any part of the epiglottis during mouth opening and tongue protrusion. The reported prevalence of Mallampati Class 0 is 0.7%–1.7% in adults [5, 6], indicating that although uncommon, this finding has been recognized as a normal anatomical variant in some individuals.

In the present case, the sustained visibility of the epiglottis for several tens of seconds during tongue protrusion suggests a persistent anatomical change rather than a transient reflex. One possible explanation is age‐related decline in the muscular support of the tongue base. Although tongue protrusion was preserved, suggesting relatively maintained function of the anterior genioglossus muscle, age‐related decline in the posterior tongue and suprahyoid muscles may have reduced structural support of the tongue base, allowing the epiglottis to become visible during tongue protrusion. Previous MRI, ultrasonography, and anatomical studies have reported age‐related fatty degeneration, volume loss, and anatomical changes involving the posterior tongue musculature and its supporting structures [7, 8, 9, 10, 11]. These findings provide a possible anatomical basis for our hypothesis; however, no objective assessment of tongue muscle morphology or swallowing function was performed in the present case.

Furthermore, the yellowish appearance on the left side of the epiglottis may represent age‐related tissue changes. Histological studies have shown that the epiglottis undergoes age‐related structural changes, including fatty degeneration and alterations in cartilage composition, which may reduce its flexibility and swallowing function [3, 12]. However, these histological changes could not be confirmed in the present case.

An important aspect of this case is that aspiration pneumonia developed 3 months after documentation of the visible epiglottis, despite the patient maintaining oral intake and having only a borderline RSST result [4]. Although this temporal association raises the possibility that the visible epiglottis was associated with early swallowing dysfunction, a causal relationship cannot be established from a single case. Furthermore, multiple factors, including advanced age, previous cerebral infarction, and frailty, may also have contributed to the development of aspiration pneumonia. The subsequent decline in the patient's activities of daily living highlights the potential clinical importance of recognizing unusual anatomical findings during routine oral examinations.

This case suggests that a visible epiglottis may represent a clinically relevant finding during routine oral examination in older adults. Recognition of this finding may prompt further assessment of swallowing function and appropriate multidisciplinary evaluation when clinically indicated.

In conclusion, we report an uncommon case of a macroscopically visible epiglottis in a 94‐year‐old patient. This finding may reflect age‐related changes in tongue‐base support and may be associated with early swallowing dysfunction. Although aspiration pneumonia developed 3 months later, a causal relationship cannot be established from a single case. Nevertheless, awareness of this finding may help dental professionals identify older adults who could benefit from further swallowing assessment and appropriate follow‐up.

## Author Contributions

**Norio Horie:** writing – review and editing, project administration, conceptualization, supervision. **Shuto Mochizuki:** supervision, writing – original draft. **Kenta Ushikubo:** writing – original draft, supervision. **Miki Yamada:** writing – original draft, conceptualization. **Shunsuke Hino:** data curation, writing – original draft, writing – review and editing. **Takahiro Kaneko:** writing – review and editing, project administration, conceptualization, supervision. **Yosuke Iijima:** data curation, writing – original draft.

## Funding

The authors have nothing to report.

## Consent

Written informed consent was obtained from the patient to publish this report in accordance with the journal's patient consent policy.

## Conflicts of Interest

The authors declare no conflicts of interest.

## Data Availability

The data that support the findings of this study are available on request from the corresponding author. The data are not publicly available due to privacy or ethical restrictions.

## References

[1] J. R. Ashford , “Impaired Oral Health: A Required Companion of Bacterial Aspiration Pneumonia,” Frontiers in Rehabilitation Sciences 5 (2024): 1337920. 38894716 10.3389/fresc.2024.1337920 PMC11183832

[2] S. Khadka , S. Khan , A. King , L. R. Goldberg , L. Crocombe , and S. Bettiol , “Poor Oral Hygiene, Oral Microorganisms and Aspiration Pneumonia Risk in Older People in Residential Aged Care: A Systematic Review,” Age and Ageing 50, no. 1 (2021): 81–87. 32677660 10.1093/ageing/afaa102

[3] M. Serikawa , K. Ambe , and A. Usami , “Histological Observations of Age‐Related Changes in the Epiglottis Associated With Decreased Deglutition Function in Older Adults,” Anatomy & Cell Biology 56, no. 3 (2023): 374–381. 37258424 10.5115/acb.23.081 PMC10520849

[4] M. Nakamori , E. Imamura , Y. Maetani , et al., “Prospective Observational Study for the Comparison of Screening Methods Including Tongue Pressure and Repetitive Saliva Swallowing With Detailed Videofluoroscopic Swallowing Study Findings in Patients With Acute Stroke,” Journal of the American Heart Association 13, no. 3 (2024): e032852. 38293925 10.1161/JAHA.123.032852 PMC11056116

[5] T. Ezri , R. D. Warters , P. Szmuk , et al., “The Incidence of Class ‘Zero’ Airway and the Impact of Mallampati Score, Age, Sex, and Body Mass Index on Prediction of Laryngoscopy Grade,” Anesthesia and Analgesia 93, no. 4 (2001): 1073–1075. 11574386 10.1097/00000539-200110000-00055

[6] E. Sepúlveda Haro , A. Raigón Ponferrada , M. Ramírez Aliaga , M. Galache Laza , J. L. Guerrero Orriach , and J. C. Mañas , “Mallampati Class Zero Airway: A Narrative Review,” Minerva Anestesiologica 88, no. 5 (2022): 390–395. 34636224 10.23736/S0375-9393.21.15945-0

[7] Y. Nakao , T. Yamashita , K. Honda , et al., “Association Among Age‐Related Tongue Muscle Abnormality, Tongue Pressure, and Presbyphagia: A 3D MRI Study,” Dysphagia 36, no. 3 (2021): 483–491. 32743742 10.1007/s00455-020-10165-4

[8] K. Hara , H. Tohara , K. Kenichiro , et al., “Association Between Tongue Muscle Strength and Masticatory Muscle Strength,” Journal of Oral Rehabilitation 46, no. 2 (2019): 134–139. 30353915 10.1111/joor.12737

[9] N. Machida , H. Tohara , K. Hara , et al., “Effects of Aging and Sarcopenia on Tongue Pressure and Jaw‐Opening Force,” Geriatrics & Gerontology International 17, no. 2 (2017): 295–301. 26800427 10.1111/ggi.12715

[10] T. J. Glass , J. E. Figueroa , J. A. Russell , B. N. Krekeler , and N. P. Connor , “Progressive Protrusive Tongue Exercise Does Not Alter Aging Effects in Retrusive Tongue Muscles,” Frontiers in Physiology 12 (2021): 740876. 34744782 10.3389/fphys.2021.740876 PMC8567011

[11] K. Kitamura , T. Watanabe , M. Yamamoto , et al., “A Newly Discovered Tendon Between the Genioglossus Muscle and Epiglottic Cartilage Identified by Histological Observation of the Pre‐Epiglottic Space,” Dysphagia 38, no. 1 (2023): 315–329. 35678869 10.1007/s00455-022-10469-7 PMC9873719

[12] C. Azuma , S. Tohno , P. Mahakkanukrauh , et al., “Age‐Dependent Increases of Calcium and Phosphorus in Human Epiglottal Cartilage,” Biological Trace Element Research 105, no. 1–3 (2005): 59–70. 16034154 10.1385/BTER:105:1-3:059

